# Supplementary material for: Isolation, Purification, and Structural Characterization of Polysaccharides from Codonopsis pilosula and Their Anti-Tumor Bioactivity by Immunomodulation
Source: Pharmaceuticals (Basel). 2023 Jun 19;16(6):895. doi: 10.3390/ph16060895 (PMC10303390; doi:10.3390/ph16060895)
Supplement: Supplementary file 1 [file pharmaceuticals-16-00895-s001.zip › pharmaceuticals-2375527-supplementary.pdf]

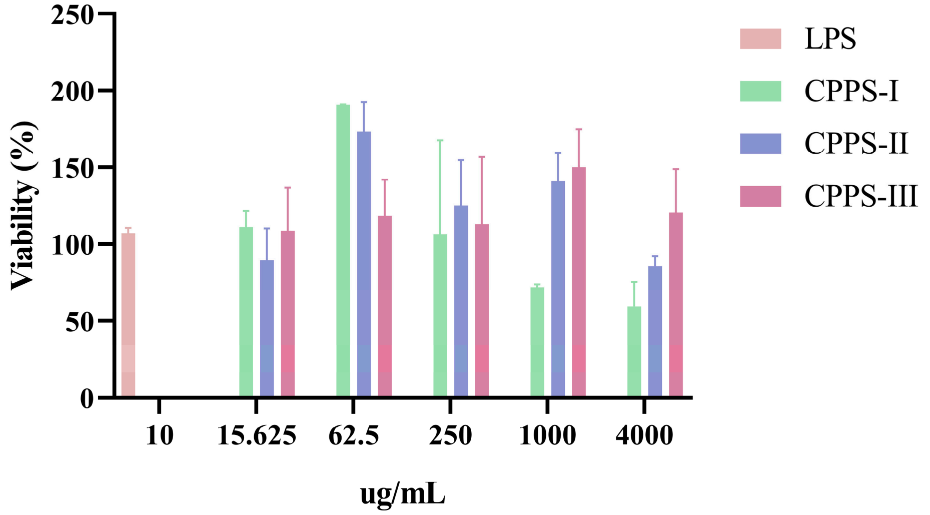

Figure S1. The viability of RAW264.7 cell cultured with different dosages of CPPS-Ⅰ,CPPS-Ⅱ， CPPS-Ⅲ and LPS evaluated by CCK-8 assay after incubation for 24h (n=4).

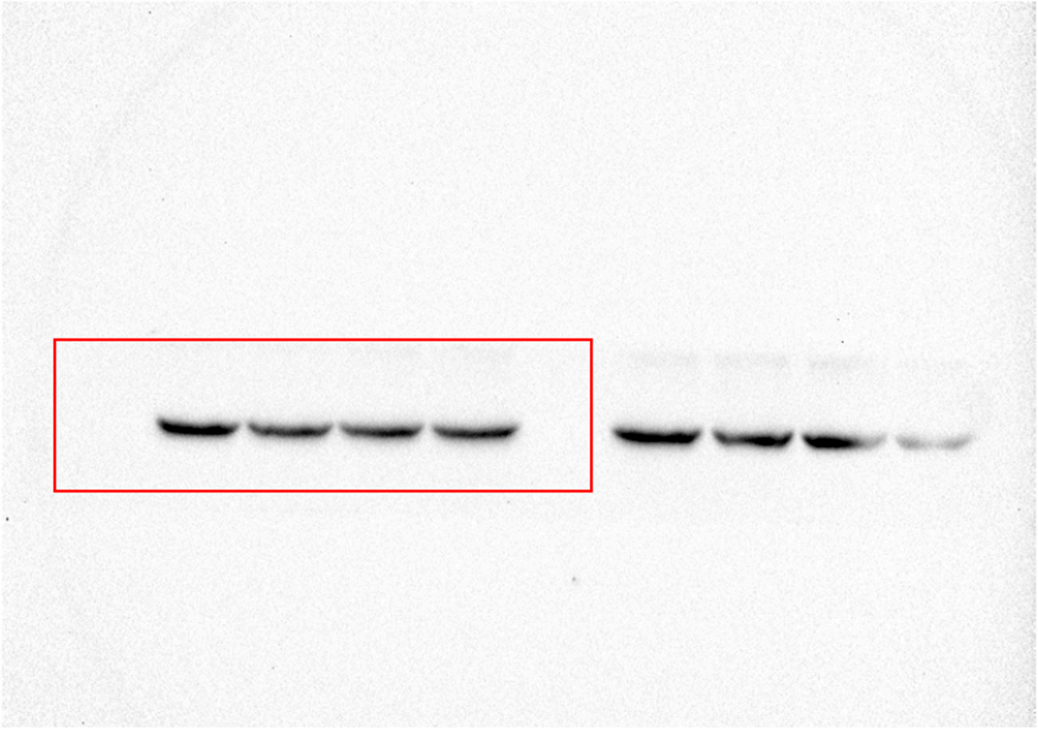

Figure S2. Beta-actin protein.

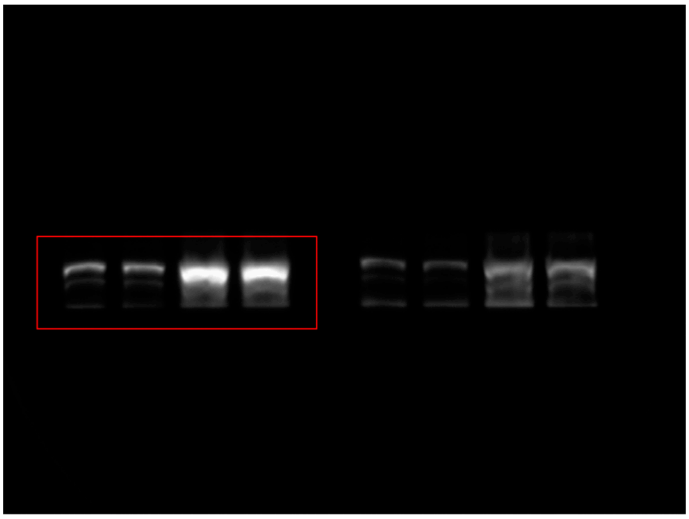

Figure S3. P-Stat1 protein expression levels of tumor tissues in different groups.

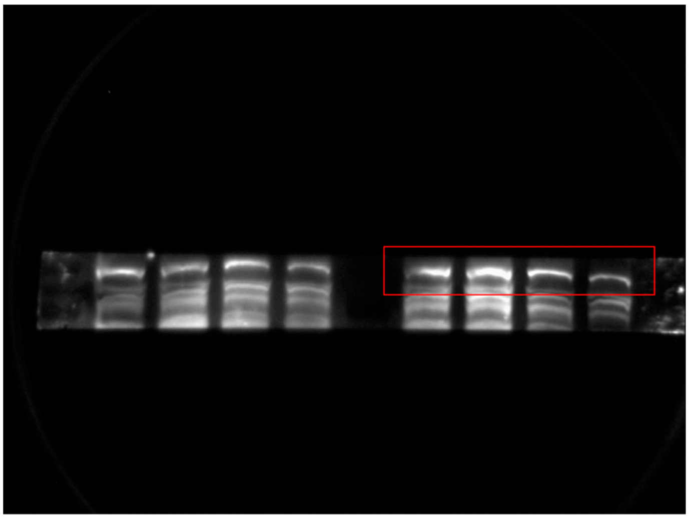

Figure S4. P-Stat3 protein expression levels of tumor tissues in different groups.

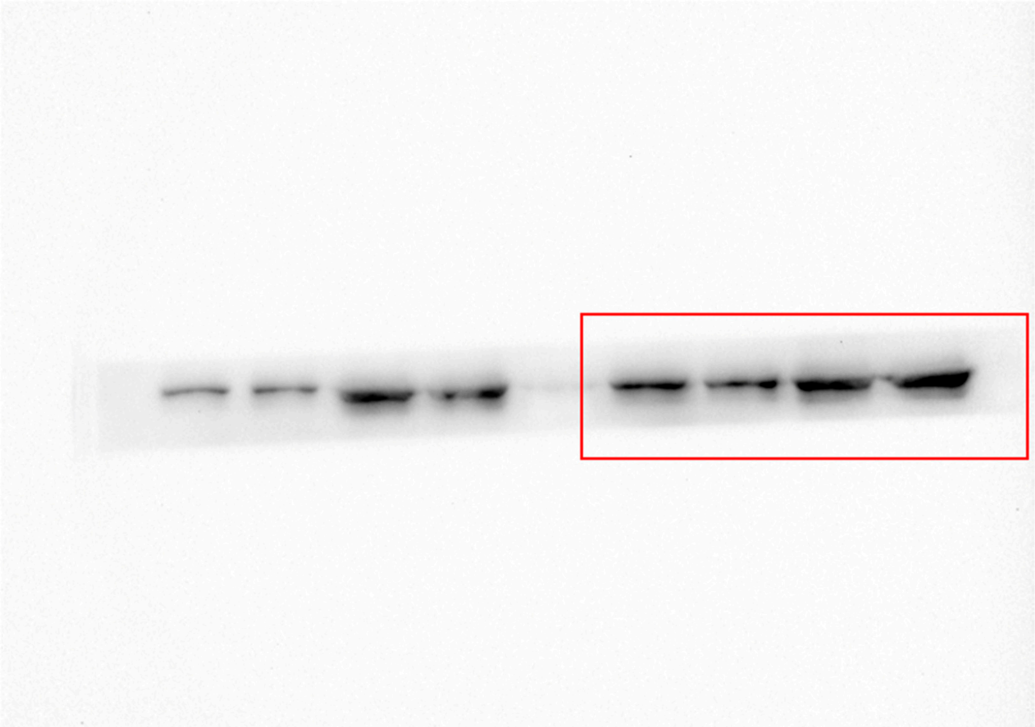

Figure S5. Stat1 protein expression levels of tumor tissues in different groups.

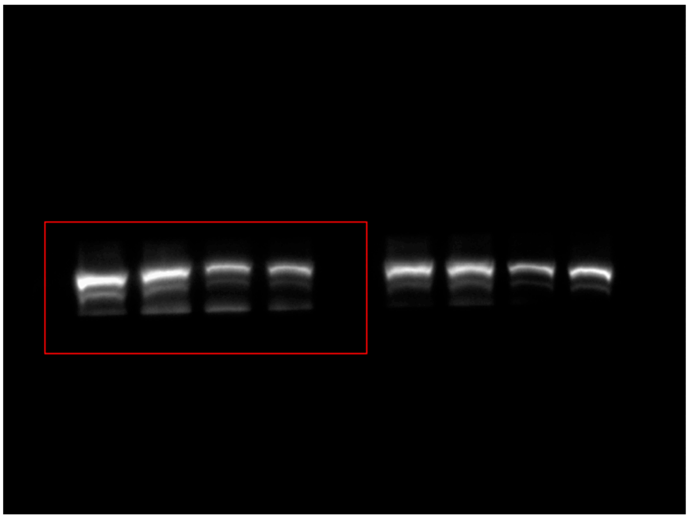

Figure S6. Stat3 protein expression levels of tumor tissues in different groups.
